# Induction of distinct plant cell death programs by secreted proteins from the wheat pathogen *Zymoseptoria tritici*

**DOI:** 10.1038/s41598-022-22660-9

**Published:** 2022-10-25

**Authors:** Thomas Welch, Carlos Bayon, Jason J. Rudd, Kostya Kanyuka, Graeme J. Kettles

**Affiliations:** 1 Birmingham Institute of Forest Research, University of Birmingham, Edgbaston, Birmingham, B15 2TT UK; 2 School of Biosciences, University of Birmingham, Edgbaston, Birmingham, B15 2TT UK; 3 Wheat Pathogenomics Team, Rothamsted Research, Harpenden, Hertfordshire, AL5 2JQ UK; 4 Cambridge Crop Research, National Institute of Agricultural Botany (NIAB), 93 Lawrence Weaver Road, Cambridge, CB3 0LE UK

**Keywords:** Biochemistry, Computational biology and bioinformatics, Molecular biology, Plant sciences

## Abstract

Cell death processes in eukaryotes shape normal development and responses to the environment. For plant–microbe interactions, initiation of host cell death plays an important role in determining disease outcomes. Cell death pathways are frequently initiated following detection of pathogen-derived molecules which can lead to resistance or susceptibility to disease depending on pathogen lifestyle. We previously identified several small secreted proteins (SSPs) from the wheat-infecting fungus *Zymoseptoria tritici* that induce rapid cell death in *Nicotiana benthamiana* following Agrobacterium-mediated delivery and expression (agroinfiltration). Here we investigated whether the execution of host cells was mechanistically similar in response to different *Z. tritici* SSPs. Using RNA sequencing, we found that transient expression of four *Z. tritici* SSPs led to massive transcriptional reprogramming within 48 h of agroinfiltration. We observed that distinct host gene expression profiles were induced dependent on whether cell death occurs in a cell surface immune receptor-dependent or -independent manner. These gene expression profiles involved differential transcriptional networks mediated by WRKY, NAC and MYB transcription factors. In addition, differential expression of genes belonging to different classes of receptor-like proteins and receptor-like kinases was observed. These data suggest that different *Z. tritici* SSPs trigger differential transcriptional reprogramming in plant cells.

## Introduction

Multicellular organisms frequently sacrifice individual cells during specific developmental stages or in response to environmental cues. Forms of programmed cell death (PCD) such as apoptosis and autophagy are a normal part of growth and development and also contribute towards the recycling of nutrients. In flowering plants, initiation of PCD plays important roles in processes as diverse as temperature stress, hypoxia, organ development and response to biotic stimuli^1–3^.

In plant-pathogen interactions, cell death is an essential part of the plant immune system^2^. In interactions with biotrophic pathogens, the active triggering of host cell death termed the hypersensitive response (HR) is often associated with disease resistance^4^. HR is considered an orderly form of PCD, characterised by DNA laddering, organelle fragmentation and cell shrinkage^5–7^. Induction of HR is assumed to both deny a nutrient supply and spatially restrict invading pathogens^8^. Activation of HR is mediated by plant disease resistance (R) proteins directly recognising secreted pathogen virulence proteins (effectors) or through recognition of effector action on other host proteins^9–11^ In contrast to HR, necrosis or other uncontrolled forms of cell death are often beneficial to pathogens. Necrotic tissue is characterised by the rupture of plasma membrane and release of cytoplasm to the extracellular spaces. Necrotrophic pathogens in particular benefit from the release of nutrients during necrosis. The importance of control of cell death is illustrated by the variety of pathogen-produced molecules which interfere with these processes. Biotrophic or hemibiotrophic pathogens frequently secrete effectors that are able to suppress activation of immune stimulation that may lead to HR^12^. In contrast, necrotrophic pathogens produce necrotrophic effectors that actively trigger host cell death pathways^13^. The lifestyle of each pathogen determines whether induction of cell death has a beneficial or detrimental outcome to the host.

The ascomycete fungus *Zymoseptoria tritici* (*Z. tritici*) causes Septoria tritici blotch (STB) disease of wheat (*Triticum aestivum*) and is a major threat to wheat productivity globally^14^. *Z. tritici* is hemibiotrophic, with infection typically being symptomless for 10–14 days, before a rapid transition to necrotrophic phase of the life cycle^15^. This is initially characterised by leaf chlorosis, followed by the appearance of necrotic lesions in infected areas and sometimes even death of infected leaves. There is considerable transcriptional reprogramming both in host plants and in the fungal cells during infection^16–18^. Wheat responses are characterised by downregulation of defence-related genes during the early symptomless phase, followed by upregulation of many of the same genes during the transition to necrotrophy^16,17^. In the fungus, there is upregulation of numerous secreted proteins that are likely to function as effectors and in genes associated with production of secondary metabolites^16,17^.

In previous work, we identified > 100 *Z. tritici* small secreted proteins (SSPs) that were upregulated during the switch from symptomless to necrotrophic growth^16^. These were classed as candidate effectors that might be involved in the induction of cell death during this transition. We used the model plant *Nicotiana benthamiana* to identify a number of SSPs with ability to induce macroscopic cell death in leaves^19,20^. We found that 13 SSPs induced cell death, and that for 12 of these, initiation of cell death required protein localisation to the apoplastic space. Further, for a smaller group of SSPs (Zt9, Zt11, Zt12) we showed that cell death required the Brassinosteroid Insensitive 1 (BRI1)‐Associated Receptor Kinase 1 (BAK1) and Suppressor of BIR1‐1 (SOBIR1) receptor-like kinases (RLKs). Both BAK1 and SOBIR1 are important co-receptors for the initiation of intracellular signalling following perception of extracellular ligands. These ligands are frequently microbe-associated molecular patterns (MAMPs) or apoplastic effectors. This indicated that initiation of cell death in response to the *Z. tritici* SSPs occurs at the cell-surface and is likely dependent on recognition by currently unidentified cell-surface immune receptors. In contrast, Zt6 was identified as a ribonuclease toxin that initiates cell death independent of BAK1/SOBIR1^20^. Moreover, Zt6 induced cell death irrespectively of whether it was secreted to the apoplast or localised to the cytoplasm. Zt6 was demonstrated to have RNase activity against rRNA and display a broad toxicity against monocot and dicot plants, yeast and bacteria, though not to *Z. tritici* itself^20^.

Based on previous results, we hypothesised that *Z. tritici* effectors may trigger different immune pathways in *N. benthamiana* that ultimately lead to macroscopically similar cell death phenotypes. To test this hypothesis, we used a transcriptomic approach (RNA sequencing, RNA-seq) to investigate early host responses to transient expression of a group of previously described *Z. tritici* SSPs that induce cell death in either a BAK1/SOBIR1-dependent or -independent manner.

## Results

### RNAseq overview

We aimed to determine the changes in the *N. benthamiana* transcriptome that occur preceding cell death driven by non-host recognition of three (Zt9, Zt11, and Zt12) *Z. tritici* SSPs, and a secreted phytotoxic RNase (Zt6) in comparison to a green fluorescent protein (GFP) control. GFP, Zt6 and SSPs were transiently expressed in *N. benthamiana* leaves using agroexpression and samples were collected at 24- and 48-h post-inoculation (hpi), i.e. prior to the HR becoming visible by eye. RNA was extracted from treated leaves to produce 30 RNA-seq libraries (five treatments x three biological replicates x two timepoints) for sequencing by paired-end sequencing on the Illumina Hiseq 2000 platform. The libraries contained 23.95–35.42 million raw reads. Subsequent quality filtering reduced the number of reads in each library by 37.4% to 51.15%. Of the remaining reads, 92.8% to 98.9% were successfully mapped to the reference *N. benthamiana* genome^21^ (Table 1).

**Table 1:** Overview of the RNA-seq data collected for each treatment.

Treatment	Replicate	No. raw reads	No. reads post-filtering	Mapped reads	% alignment rate
Green fluorescent protein 24 h	1	29,632,800	15,043,938	14,602,207	98.59
Green fluorescent protein 24 h	2	28,034,184	16,374,738	15,431,491	95.73
Green fluorescent protein 24 h	3	27,135,887	13,275,593	12,882,194	98.56
Green fluorescent protein 48 h	1	30,478,092	18,756,098	17,567,294	95.26
Green fluorescent protein 48 h	2	30,961,071	16,482,238	15,930,691	98.34
Green fluorescent protein 48 h	3	23,947,546	14,644,113	13,453,249	93.53
Zt6 24 h	1	27,366,516	16,894,171	16,337,539	98.38
Zt6 24 h	2	29,777,551	18,459,699	17,046,698	93.79
Zt6 24 h	3	32,521,194	20,013,222	19,366,353	98.34
Zt6 48 h	1	26,459,619	15,042,329	13,746,564	92.83
Zt6 48 h	2	28,336,919	17,480,929	16,911,429	98.40
Zt6 48 h	3	31,071,683	18,719,718	17,363,364	94.21
Zt9 24 h	1	29,426,692	15,565,703	15,122,756	98.76
Zt9 24 h	2	27,278,801	15,849,440	14,386,160	92.20
Zt9 24 h	3	32,763,879	16,106,357	15,677,521	98.83
Zt9 48 h	1	33,367,108	20,400,605	18,581,986	92.58
Zt9 48 h	2	27,783,195	14,402,672	13,992,594	98.68
Zt9 48 h	3	24,128,224	14,765,579	13,204,618	90.88
Zt11 24 h	1	25,960,835	13,004,520	12,668,292	98.89
Zt11 24 h	2	28,971,906	17,874,511	17,309,244	98.52
Zt11 24 h	3	24,640,400	12,036,157	11,725,777	98.91
Zt11 48 h	1	25,904,899	14,658,337	14,215,966	98.54
Zt11 48 h	2	20,601,920	10,108,513	9,836,307	98.90
Zt11 48 h	3	30,912,455	18,370,079	17,786,924	98.32
Zt12 24 h	1	27,944,558	16,863,219	15,503,985	93.32
Zt12 24 h	2	26,596,304	15,083,099	14,507,692	98.15
Zt12 24 h	3	32,596,510	20,162,208	18,479,220	93.10
Zt12 48 h	1	30,851,089	18,800,164	18,208,649	98.46
Zt12 48 h	2	35,424,590	22,184,733	20,792,722	95.27
Zt12 48 h	3	32,486,965	19,870,839	19,252,930	98.51

**Figure 1:**
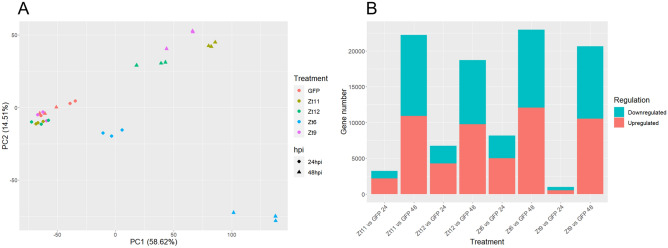
(**A**) PCA plot of RNA-seq data. SSP treatments and timepoints indicated. (**B**) Total numbers of upregulated (red) and downregulated (blue) DEGs.

Principle component analysis (PCA) of the overall gene-expression profile showed that replicates of each treatment clustered tightly, as well as revealing minimal difference between the GFP and SSP treatments at 24hpi (Fig. 1A). This pattern changed by 48hpi, with SSP treatments showing clear separation compared to the GFP control, although with minimal difference among themselves. In contrast, Zt6 expression induced a different gene-expression profile compared to both the GFP and SSP treatments at 24hpi. This was further exaggerated by the 48hpi timepoint (Fig. 1A).

To examine these different responses, differential expression analysis was conducted using DESeq2 Bioconductor package^22^, with FDR less than 0.05, to identify differentially expressed genes (DEGs) between the SSP and Zt6 treatments compared to the GFP control (Fig. 1B). At 24hpi across all treatments, more DEGs were upregulated than downregulated. At 48hpi, the ratio of upregulated and downregulated DEGs was similar, although the total number of DEGs greatly increased. The increase in number of DEGs was most noticeable for the Zt9 treatment, which had a much smaller number of DEGs at 24hpi than the other treatments (Fig. 1B).

**Figure 2:**
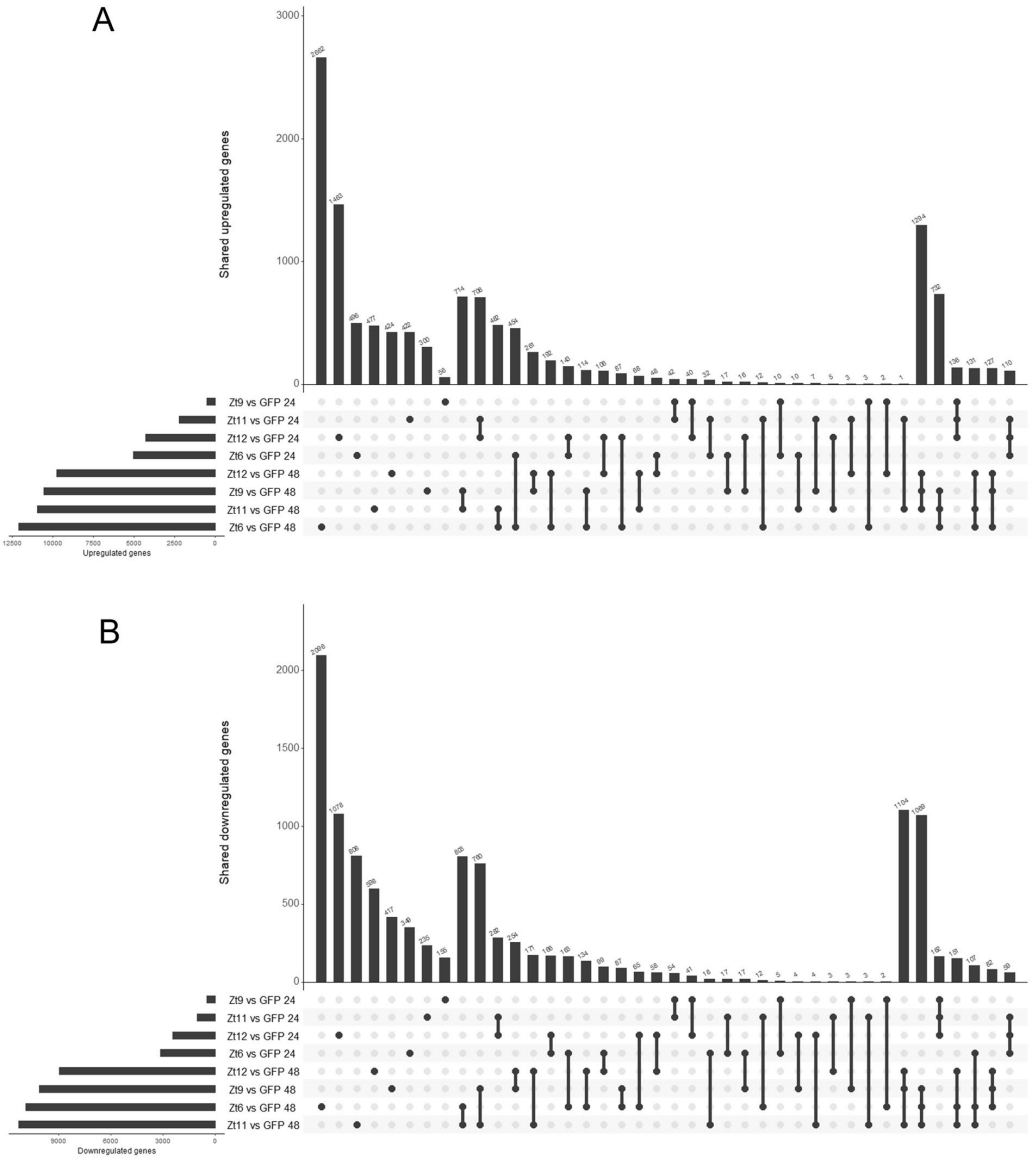
Plots showing total numbers of significantly upregulated (**A**) and significantly downregulated (**B**) DEGs. Numbers of DEGs shared among one, two, and three treatment combinations are represented by bar size in each main plot, with the individual treatments that make up each combination represented in the bead and line chart below. Total numbers of DEGs in each individual treatment are represented by bar size on the lower left of each plot. Plots generated using UpsetR^63^.

The profound difference between the Zt6 and SSP treatments observed by PCA (Fig. 1A), was reflected in the number of DEGs shared between treatments (Fig. 2). For example, the number of upregulated genes common to all three SSP treatments at 48 h (1294) was far higher than that of any other group of three treatments at 48hpi that contained Zt6 (732, 131, 127 DEGs respectively, Fig. 2A). A similar pattern was observed for downregulated genes, although one particular Zt6-containing group of treatments (Zt11/Zt9/Zt6 vs GFP) at 48 hpi shared a much larger number of DEGs (1069) than other groupings, and almost as much as the SSP treatments group (Zt9/Zt11/Zt12 vs GFP, 1104 DEGs, Fig. 1B). Noticeably, at 48 hpi the Zt11 treatment shared far more DEGs with the Zt6 treatment (482 and 808 upregulated and downregulated respectively) than either of the other two SSP treatments, but at 24hpi was much more similar to the Zt12 treatment (Fig. 1).

### Gene ontology enrichment analysis

In order to examine whether differentially expressed genes (DEGs) were involved in specific developmental processes, we performed a GO-enrichment analysis of up- and down-regulated genes for the Zt6 (Tables 2, 3) and SSP treatments (Tables 4, 5). For brevity, only the top 20 GO terms (i.e. those with the largest number of DEGs averaged across treatments) enriched in the SSP treatments are listed here.

**Table 2:** Top 20 GO terms enriched among Zt6 treatment in upregulated DEGs and their BH-FDR adjusted *P *values.

Category	Ontology	GO term	Zt6 24hpi	Zt6 48hpi
GO:0005622	CC	Intracellular	–	1.61E−16
GO:0016772	MF	Transferase activity, transferring phosphorus-containing groups	1.47E−17	0.012755501
GO:0006468	BP	Protein phosphorylation	1.05E−17	0.005818144
GO:0004672	MF	Protein kinase activity	1.24E−17	0.005818144
GO:0003824	MF	Catalytic activity	–	0.012632623
GO:0005524	MF	ATP binding	1.11E−05	–
GO:0003735	MF	Structural constituent of Ribosome	–	1.85E−22
GO:0006412	BP	Translation	–	1.85E−22
GO:0006355	BP	Regulation of transcription, DNA-templated	6.74E−09	2.65E−05
GO:0008152	BP	Metabolic process	–	0.005084615
GO:0005840	CC	Ribosome	–	1.57E−21
GO:0004674	MF	Protein serine/threonine kinase activity	3.08E−18	2.34E−06
GO:0005515	MF	Protein binding	0.001049327	–
GO:0003700	MF	DNA-binding Transcription factor activity	8.46E−20	4.24E−07
GO:0043565	MF	Sequence-specific DNA binding	5.52E−11	8.73E−05
GO:0016787	MF	Hydrolase activity	–	0.012631703
GO:0050660	MF	Flavin adenine dinucleotide binding	–	0.011249318
GO:0003743	MF	Translation initiation factor activity	–	0.026946629
GO:0006096	BP	Glycolytic process	–	0.001674608
GO:0004842	MF	Ubiquitin–protein transferase activity	0.000193821	–

**Table 3:** Top 20 GO terms enriched among Zt6 treatment in downregulated DEGs and their BH-FDR adjusted *P *values.

Category	Ontology	GO term	Zt6 24hpi	Zt6 48hpi
GO:0055114	BP	Oxidation–reduction process	–	4.85E−05
GO:0016020	CC	Membrane	–	3.38E−06
GO:0003824	MF	Catalytic activity	–	0.000329751
GO:0008152	BP	Metabolic process	–	4.85E−07
GO:0016491	MF	Oxidoreductase activity	–	1.29E−05
GO:0005975	BP	Carbohydrate metabolic process	–	2.66E−05
GO:0006508	BP	Proteolysis	–	0.003108672
GO:0005737	CC	Cytoplasm	–	0.019343795
GO:0015979	BP	Photosynthesis	–	2.84E−08
GO:0005509	MF	Calcium ion binding	–	0.047329562
GO:0045454	BP	Cell redox homeostasis	–	0.019313876
GO:0009765	BP	Photosynthesis, light harvesting	–	8.51E−10
GO:0009523	CC	Photosystem II	–	3.38E−06
GO:0006096	BP	Glycolytic process	–	0.017098235
GO:0004222	MF	Metalloendopeptidase activity	–	0.030923014
GO:0009654	CC	Photosystem II oxygen evolving complex	–	2.38E−07
GO:0019898	CC	Extrinsic component of membrane	–	3.38E−06
GO:0004427	MF	Inorganic diphosphatase activity	–	0.000591418
GO:0009538	CC	Photosystem I reaction center	–	0.00010569
GO:0042132	MF	Fructose 1,6-bisphosphate 1-phosphatase activity	–	0.008522977

**Table 4:** Top 20 GO terms enriched among SSP treatments in upregulated DEGs and their BH-FDR adjusted *P *values.

Category	Ontology	GO term	Zt9 24hpi	Zt11 24hpi	Zt12 24hpi	Zt9 48hpi	Zt11 48hpi	Zt12 48hpi
GO:0000166	MF	Nucleotide binding	–	–	–	–	0.007614	–
GO:0016020	CC	Membrane	–	–	0.019551	–	–	–
GO:0003723	MF	RNA binding	–	–	–	0.01373	0.001801	0.003530677
GO:0005737	CC	Cytoplasm	–	–	–	4.80E−05	0.005605	3.39E−08
GO:0006886	BP	Intracellular protein transport	–	–	–	0.025869	–	0.009031875
GO:0005525	MF	GTP binding	–	–	–	0.01373	0.033079	–
GO:0006457	BP	Protein folding	–	–	–	0.000534	0.021678	–
GO:0015031	BP	Protein transport	–	–	–	0.001189	0.020858	0.009555824
GO:0003924	MF	GTPase activity	–	–	0.017039	0.002129	–	0.012699485
GO:0005215	MF	Transporter activity	–	–	0.049319	–	–	–
GO:0006511	BP	Ubiquitin-dependent protein catabolic process	–	–	–	0.040493	0.033365	–
GO:0006184	BP	Obsolete GTP catabolic process	–	–	0.001484	0.030019	–	–
GO:0015035	MF	Protein disulfide oxidoreductase activity	–	–	4.77E−07	–	–	–
GO:0004298	MF	Threonine-type endopeptidase activity	–	–	–	3.81E−11	1.62E−07	4.28E−05
GO:0005839	CC	Proteasome core complex	–	–	–	3.81E−11	1.62E−07	4.28E−05
GO:0051603	BP	Proteolysis involved in cellular protein catabolic process	–	–	3.81E−11	1.62E−07	4.28E−05	
GO:0051536	MF	Iron-sulfur cluster binding	–	–	–	–	–	0.025876055
GO:0006913	BP	Nucleocytoplasmic transport	–	–	–	0.044736	–	–
GO:0004175	MF	Endopeptidase activity	–	–	–	7.37E−11	5.20E−06	5.71E−05
GO:0015935	CC	Small ribosomal subunit	–	–	–	–	–	0.000673305

**Table 5:** Top 20 GO terms enriched among SSP treatments in downregulated DEGs and their BH-FDR adjusted *P*-values.

Category	Ontology	GO Term	Zt9 24hpi	Zt11 24hpi	Zt12 24hpi	Zt9 48hpi	Zt11 48hpi	Zt12 48hpi
GO:0005524	MF	ATP binding	–	–	–	0.003562	0.000859	0.001602
GO:0016772	MF	Transferase activity, transferring phosphorus-containing groups	–	–	–	0.00188	0.001457	0.000473
GO:0006468	BP	Protein phosphorylation	–	–	–	0.00051	2.08E−05	0.000285
GO:0004672	MF	Protein kinase activity	–	–	–	0.000472	2.05E−05	0.000285
GO:0004674	MF	Protein serine/threonine kinase activity	–	–	–	0.046399	0.008818	–
GO:0005622	CC	Intracellular	–	–	–	0.039391	–	–
GO:0004553	MF	Hydrolase activity, hydrolyzing O-glycosyl compounds	–	–	–	0.016349	0.031375	0.000686
GO:0003676	MF	Nucleic acid binding	–	–	0.013303	–	–	–
GO:0006629	BP	Lipid metabolic process	–	–	–	0.014781	0.00668	–
GO:0007165	BP	Signal transduction	–	–	–	0.04235	–	–
GO:0008017	MF	Microtubule binding	–	–	–	0.025374	0.006388	3.70E−06
GO:0003777	MF	Microtubule motor activity	–	–	–	–	–	0.002007
GO:0007018	BP	Microtubule-based movement	–	–	–	–	–	0.002007
GO:0003924	MF	GTPase activity	–	–	–	0.025071	–	0.043564
GO:0005871	CC	Kinesin complex	–	–	–	–	0.042549	0.001638
GO:0006184	BP	Obsolete GTP catabolic process	–	–	–	0.008125	0.016356	0.001431
GO:0046982	MF	Protein heterodimerization activity	–	–	–	2.45E−07	7.58E−09	0.000252
GO:0030246	MF	Carbohydrate binding	–	–	–	0.025642	–	–
GO:0000786	CC	Nucleosome	–	–	–	5.28E−11	6.23E−08	8.46E−05
GO:0006633	BP	Fatty acid biosynthetic process	–	–	–	–	–	0.042652

Most significantly enriched GO terms were present across several treatments, and most were enriched only at the 48hpi time point. Among upregulated DEGs, only four GO terms were enriched in more than one treatment at 24hpi, whilst none were enriched among downregulated DEGs. Out of all significantly enriched GO terms (70 in downregulated DEGs and 113 in upregulated DEGs) 37 were enriched only at the 24hpi time point of only one treatment. Noticeably, 20 of these were enriched only in the Zt12 treatment, while eight, seven, and two were exclusive to the Zt11, Zt9, and Zt6 treatments respectively. Only one of the eight GO terms exclusive to Zt11 (sulfate reduction) was enriched in the downregulated DEGs.

The contrasting transcriptional response to SSP and Zt6 treatments revealed by PCA (Fig. 1A) was also observed in terms of enriched GO categories. Among the upregulated DEGs, only seven GO terms were enriched in all treatments at 48hpi (structural constituent of ribosome, translation initiation factor activity, intracellular, ribosome, translation, metabolic process, and ribosome biogenesis) (Tables 2, 4). Most other GO terms were enriched in either one or all SSP treatments, or they were exclusive of the Zt6 treatment. There were only two terms (hydrolase activity, and catalytic activity) that were common to Zt6 and at least one other treatment. This pattern was similar in downregulated DEGs, where only nine GO terms were enriched across all treatments (carbohydrate metabolic process, extrinsic component of membrane, fructose 1,6-bisphosphate 1-phosphatase activity, membrane, photosynthesis, light harvesting, photosystem I reaction centre, photosystem II, and photosystem II oxygen evolving complex) (Tables 3, 5) also in this case at 48hpi. Strikingly, these nine common GO terms included five (of a total six) photosynthesis-related GO terms. Of those remaining, only five were shared between the Zt6 treatment and at least one SSP treatment. Furthermore, whilst four photosynthesis related GO terms were also significantly enriched among upregulated DEGs, three were exclusive to the Zt11 treatment at 24hpi, and one was significantly enriched in both Zt11 and Zt12 treatments, also only at 24hpi.

The response to Zt6 expression was characterised by an overrepresentation of up-regulated genes involved in protein phosphorylation and kinase activity (Table 2). This included the GO terms transferase activity (of phosphorous-containing groups), protein phosphorylation, protein kinase activity, and protein serine/threonine kinase activity. By contrast, these signaling-related GO terms were enriched amongst downregulated genes for the SSP treatments (Table 5).

GO categories overrepresented among upregulated genes in the SSP treatments were those related to cellular protein catabolic processes. This included threonine type endopeptidase activity, endopeptidase activity, proteasome complex, and proteolysis involved in cellular catabolic process, as well as categories involved in other cellular protein metabolic processes such as protein folding (Table 4).

Over-represented among genes downregulated in the SSP treatments were those involved in diverse processes such as response to hormone and signal transduction, binding and activity of microtubules, lipid and fatty acid metabolism, DNA replication, and protein complex genes (e.g. MCM complex and kinesin complex) (Table 5). Closer investigation showed that downregulation of histone protein genes was the sole driver of enrichment of the GO term nucleosome. GTPase activity and GTP-catabolic process were the only GO terms enriched among both up and downregulated genes of the SSP treatments.

### Differential expression of immune receptor-like genes

We previously demonstrated the requirement for the cell-surface co-receptors NbBAK1 and NbSOBIR1 for full induction of cell death by the SSP effector group^19^. These co-receptors are not required for Zt6-induced cell death^20^. It is therefore likely that cell death induced by the SSP treatments is a form of immune receptor-mediated programmed cell death. In contrast, Zt6-induced cell death is likely to be more similar to necrosis. Expression of receptors is often upregulated in response to the presence of their ligand^23–25^. We therefore assessed whether there were differential expression patterns of cell surface and cytoplasmic receptor gene families commonly associated with pathogen-associated molecular pattern (PAMP) and/ or effector recognition. Lists of these gene family members used in this assessment were obtained by filtering of the *N. benthamiana* genome annotation (GFF3) file according to their description in the note field (Supplementary material 1).

**Figure 3:**
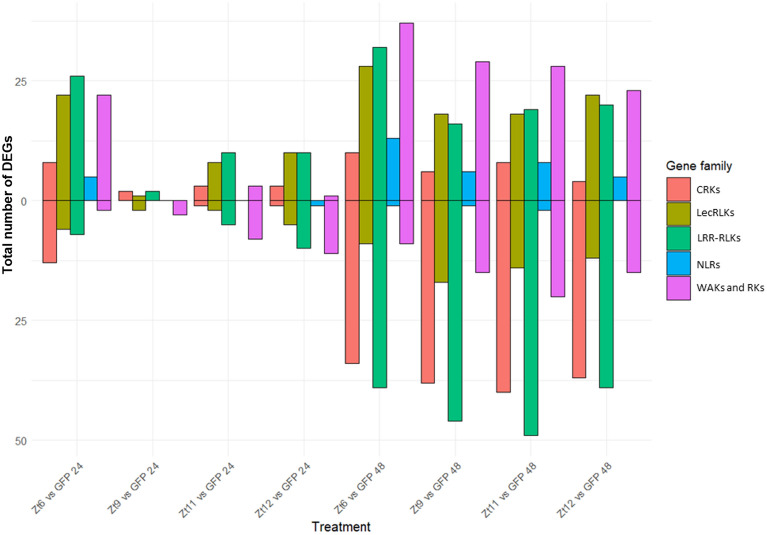
Total numbers of DEGs from each of five pathogen response associated gene families in each treatment. CRKs, LecRLKs, LRR-RLKs, NLRs, WAKs and RKs. Number of upregulated DEGs are represented by bar size above the x-axis, number of downregulated DEGs are represented by bar size below the x-axis.

There were clear differences in transcriptional profiles across receptor families induced by Zt6 and SSP treatments. At 24hpi, transcriptional changes among wall-associated kinases (WAKs), receptor like kinases (RLKs), leucine-rich repeat receptor like-kinases (LRR-RLKs), and lectin-receptor kinases (LecRLKs) showed a clear bias toward upregulation in the Zt6 treatment (Fig. 3). For WAKs and RLKs this bias was reversed in the SSP treatments, and for LRR-RLKs there was a smaller number of genes upregulated in the SSP treatments in comparison to Zt6. For LecRLKs, a similar bias towards upregulation existed between the Zt6, Zt11, and Zt12 treatments, although this was less prominent for Zt11 and Zt12. At 48hpi, LRR-RLKs in the SSP treatments showed a bias towards downregulation, this was less prominent in the Zt6 treatment. Interestingly, at 48hpi cysteine-rich receptor-like kinases (CRKs) showed a strong bias toward downregulation across all treatments. Genes annotated as nucleotide-binding site leucine-rich repeats (NLRs) showed bias toward upregulation at 48hpi across all treatments, though this was a comparatively small number of genes relative to the total *N. benthamiana* NLR gene complement.

**Figure 4:**
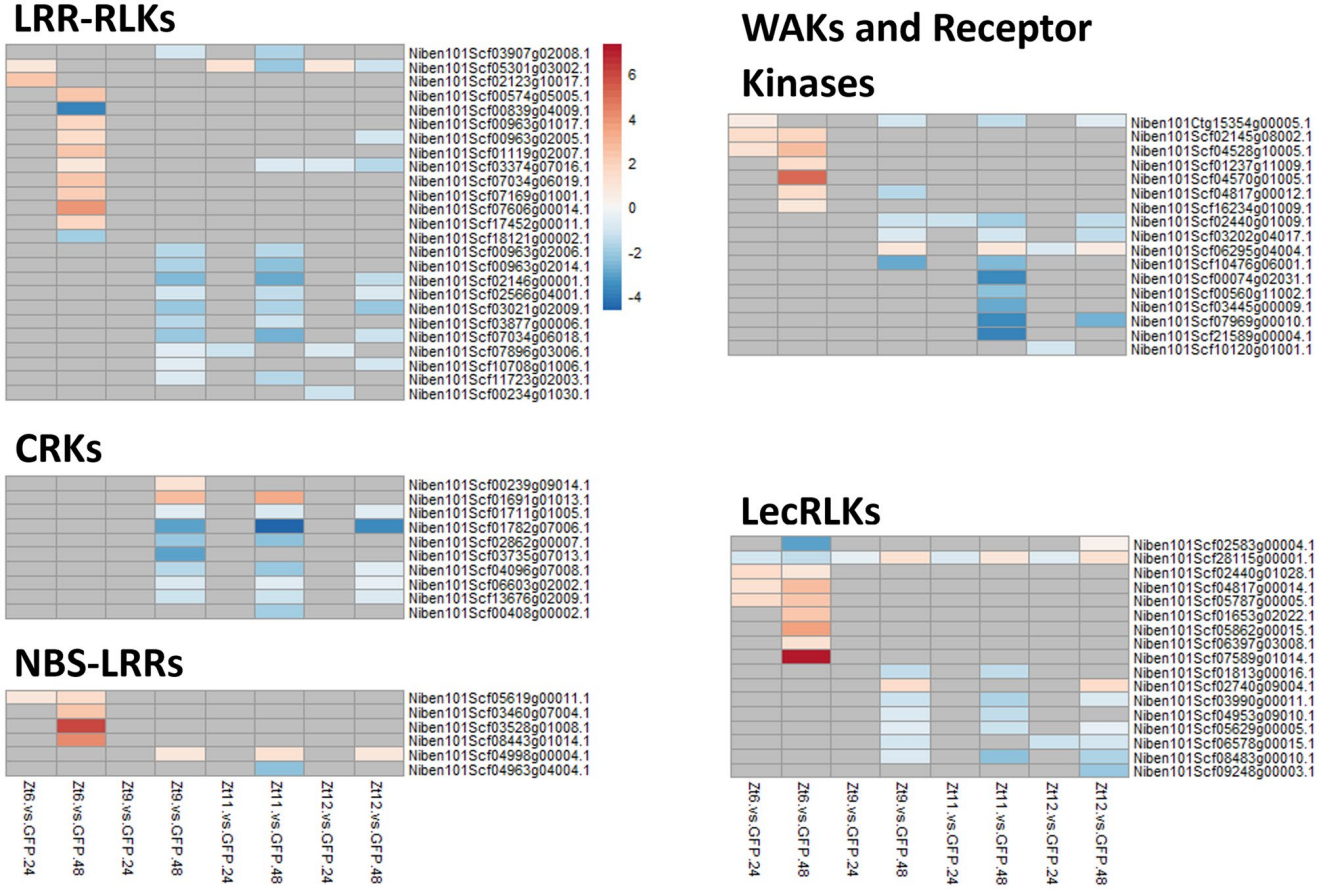
Expression profile of defence associated genes with marked difference in expression change between Zt6 and Zt9, Zt11, and Zt12.

**Figure 5:**
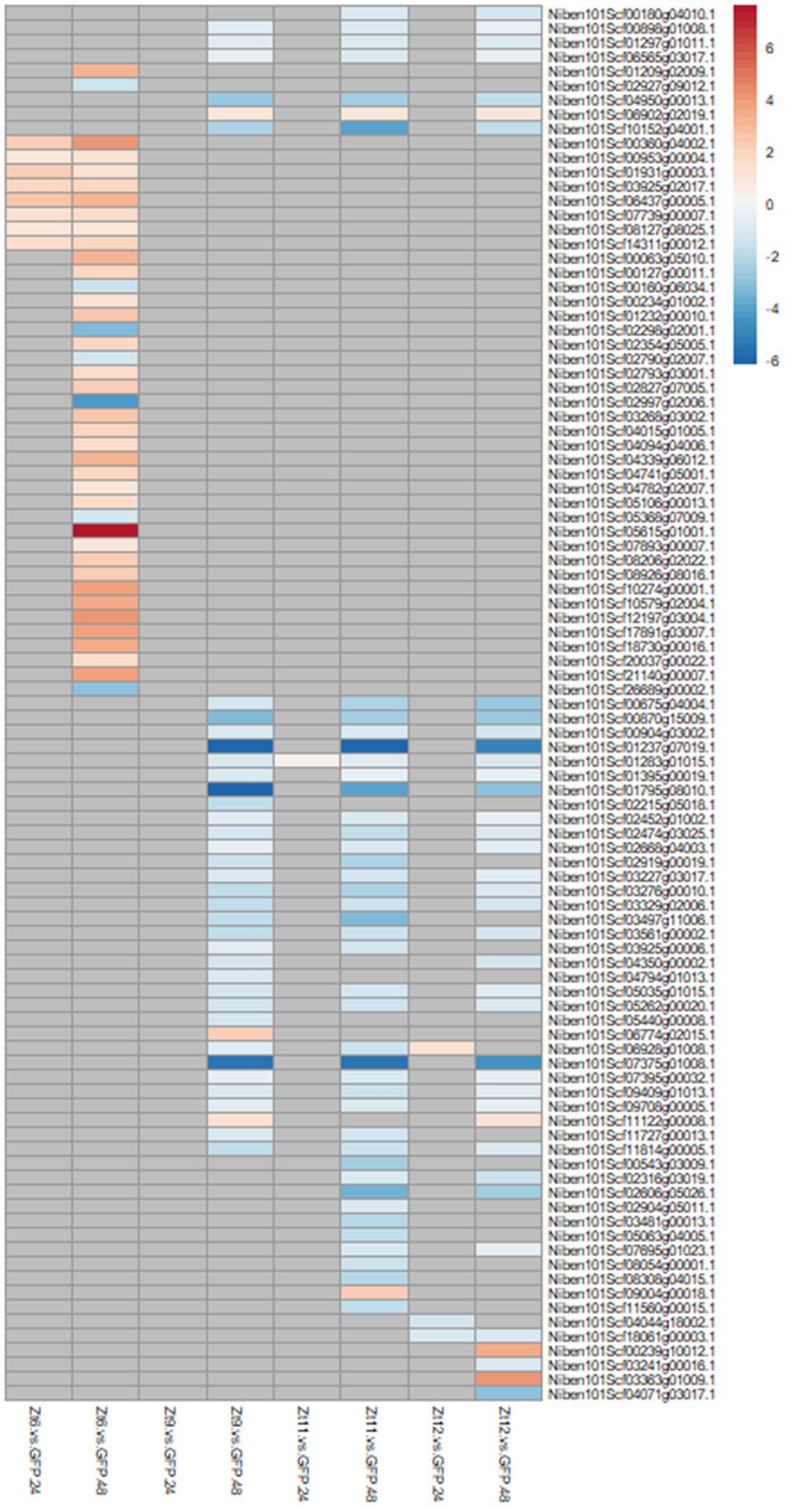
Expression profile of RLPKs genes with marked difference in expression change between Zt6 and Zt9, Zt11, and Zt12.

Given the differences between the transcriptomes of the Zt6 and SSP treatments, we next identified and investigated specific genes within these five receptor gene families, based on whether they showed a marked difference in expression change between the Zt6 and SSP treatments. For the purposes of this investigation, we defined a “marked difference” as a log_2_ fold change of an absolute value of at least 1.0 in one or all of the SSP treatments which was either not present or reversed in the Zt6 treatment, or a log_2_ fold change of an absolute value of at least 1.0 in the Zt6 treatment which was either not present or reversed across all the SSP treatments. For all six receptor gene families, few expression changes were apparent at 24hpi in comparison to the GFP control. However, at 48hpi a clear difference in expression pattern is visible between the Zt6 and SSP treatments. Zt6 treatment specifically induced expression of 11 out of a total 149 LRR-RLKs, 4 out of a total 65 NLRs, 7 out of a total 107 WAKs/RLKs and 7 out of a total 54 LecRLKs in the current *N. benthamiana* genome annotation (Fig. 4). Zt6 also induced expression of 34 out of a total 795 RLPKs in the current *N. benthamiana* genome annotation (Fig. 5). Only two LRR-RLKs, two LecRLKs and 7 RLPKs were downregulated in response to Zt6 treatment (Fig. 4). Interestingly, no CRKs showed any transcriptional response to Zt6 (Fig. 4). Very few receptor genes were induced by the SSP treatments, although notable exceptions included one NLR gene induced by all three SSP treatments at 48hpi (Fig. 4), and two RLPK genes relatively strongly induced only by the Zt12 treatment at 48hpi (Fig. 5). Most receptor family genes that were transcriptionally responsive to the SSP treatments were downregulated. These included a notable over-abundance of WAKs and RLKs that were strongly downregulated in response only to the Zt11 treatment at 48hpi. Together, this suggests that there is reprogramming of receptor gene expression following exposure to SSPs, and that this differs between individual SSPs.

### Differential expression of transcription factors, senescence- and programmed cell death-associated genes

**Figure 6:**
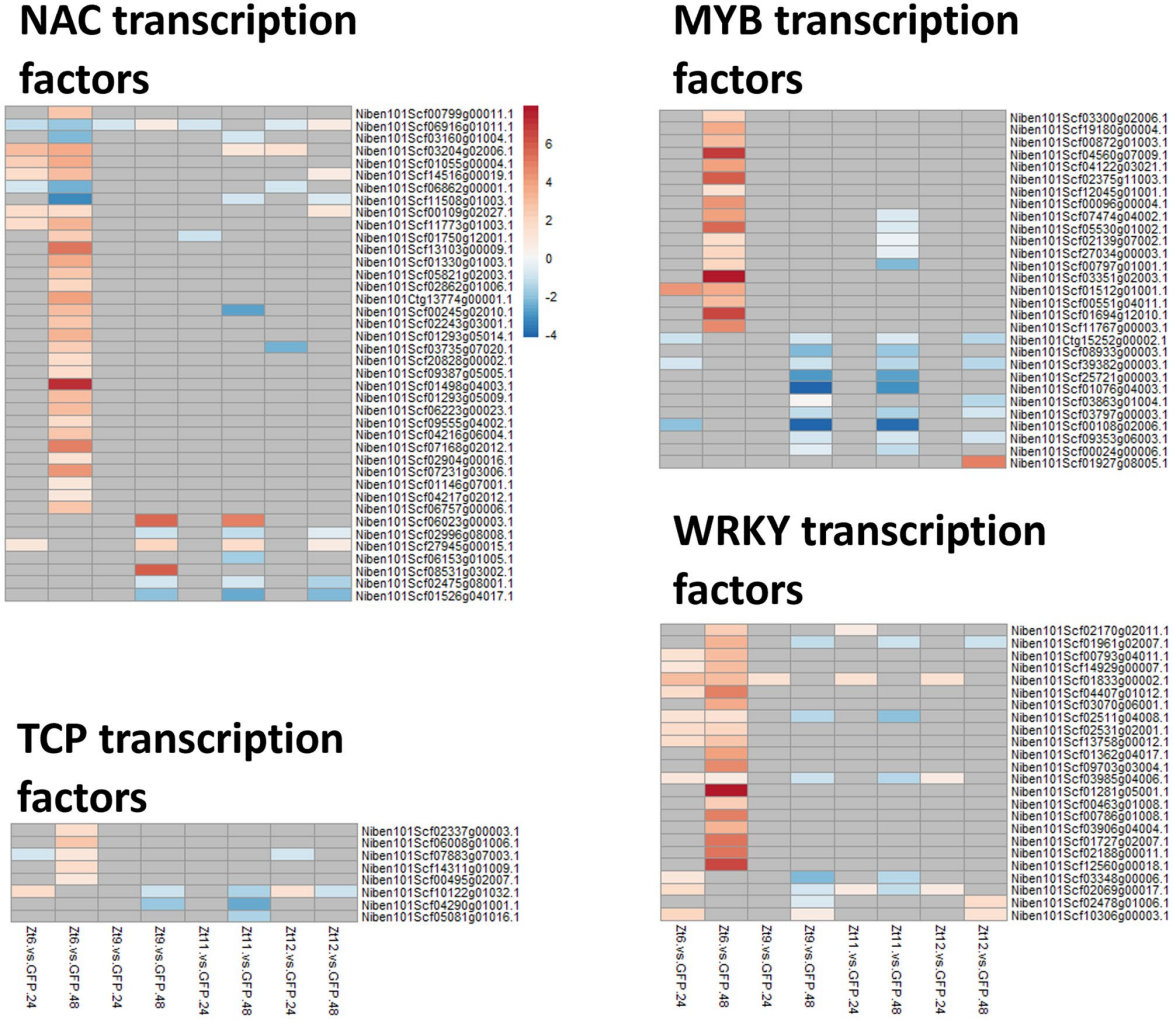
Expression profile of senescence regulating transcription factor genes with marked difference in expression change between Zt6 and Zt9, Zt11, and Zt12.

There are differences in how developmental, pathogen-associated, and stress-induced PCD is executed. These distinct but partially overlapping pathways share some common components, although no pathway is fully characterised^26–28^. Our GO enrichment analysis showed that Zt6 and SSP treatments appear to be inducing different types of transcriptional reprogramming, and that Zt6-induced cell death is distinct from ordered receptor-mediated PCD^26^. We therefore investigated whether gene families commonly associated with transcriptional reprogramming related to senescence and PCD displayed differential expression patterns between the Zt6 and SSP effector treatments. We first evaluated the expression of the NAC, WRKY, TCP and MYB transcription factor (TF) families (Fig. 6), which are widely reported as regulators of leaf senescence^29–32^. At 24hpi there were few differential expression changes across any of the treatments. However, by 48hpi there were clear differences in expression patterns between Zt6 and SSP treatments. Overall, Zt6 treatment induced expression of 29 out of a total 241 NACs, 20 out of a total 151 WRKYs, 5 out of a total 61 TCPs and 18 out of a total 242 MYBs in the current *N. benthamiana* genome annotation. Only four TFs (all NAC family) were downregulated in response to Zt6 at this timepoint. In contrast, few TFs of any family were induced by the SSP treatments. Indeed, for WRKY, MYB and TCP families the majority of differentially expressed TFs were downregulated in response to effector treatment. There was minimal overlap between the individual genes transcriptionally induced by Zt6, and those transcriptionally repressed by the SSP treatments.

Several other non-TF gene families have previously been linked to senescence and cell death in plants. Metacaspases, zinc-finger domain containing proteins and HR-inducing proteins have all been shown to regulate PCD or HR^33,34^. The senescence-associated genes *SAG1* (*Arabidopsis thaliana*), *SAG102* (*Medicago truncatula*), *phytoalexin–deficient 4* (*PAD4/SAG101*) and *harpin-induced gene 1* (*HIN1*), are markers of senescence and HR respectively^35–37^. In addition, *STAY-GREEN* (*SGR*) genes operate downstream of NAC TF regulation of senescence in the catabolism of chlorophyll^38^. We investigated expression of these genes across all treatments and found that expression of genes encoding metacaspases, SAG, HR-inducing and SGR proteins between Zt6 and SSP treatments was similar (Fig. S1). However, a zinc-finger containing protein encoding gene was strongly induced by Zt6 treatment at 24- and 48hpi but not by the SSP treatments.

## Discussion

In this investigation, we aimed to understand the mechanisms of cell death induced by several SSPs of *Z. tritici* in a non-host plant *N. benthamiana*. To do this, we made use of the Agrobacterium-mediated transient expression system originally used to identify the cell death-inducing activity of these proteins. Our results indicate that expression of this group of proteins initiate massive transcriptional reprogramming of plant cells prior to onset of macroscopic cell death. Furthermore, the cytotoxic secreted ribonuclease Zt6 induces a transcriptional response distinct from other SSPs.

The transcriptional responses to SSP treatments were broadly similar, and clearly distinct from that induced by Zt6 expression. PCA (Fig. 1A) illustrates clustering of SSP treatments at 24hpi and obvious separation from both Zt6 and GFP control treatments. This separation is further exaggerated by 48hpi. The total number of genes that are differentially expressed is greater in Zt6 treated leaves at 24hpi in comparison to SSP treatments. This indicates that transcriptional reprogramming begins earlier for Zt6 compared to SSP treatments. This is consistent with the earlier onset of macroscopic cell death induced by Zt6 in comparison with the SSPs^20^. This may reflect that Zt6 induces cell death due to its enzymatic RNase activity targeting rRNA^20^, whereas the SSPs presumably induce immune receptor-mediated cell death^19^.

Given the dependency of SSP induced cell death on BAK1, broad similarity of transcriptional response to all three SSP treatments is not surprising. BAK1 functions as a co-receptor for various RLKs, including some whose ligands are PAMPs or secreted effectors; it is a convergence point of multiple pathogen-triggered physiological pathways that lead to PCD^39^. However, BAK1 dependent PCD has been shown to be highly ligand specific and could still proceed via a variety of mechanisms, dependent upon how its PAMP/effector co-receptor role disrupts its normal functioning and modifies how it subsequently interacts with other RLKs (including but not limited to BIR1 and SOBIR1), which also have important roles in regulation of PCD^39^.

A notable difference in response to the Zt6 and SSP treatments was differential regulation of genes involved in microtubule activity, movement and binding. Downregulation of genes in these categories was enriched in SSP treatments at 48hpi (Table 5), but not in Zt6 at either timepoint (Table 3). Depolymerisation of the microtubule network has been associated with PCD^40^, in particular in HR reactions in *A. thaliana*^41^ and soybean^42^. Microtubule reorganisation is also associated with developmental PCD processes such as self-incompatibility^43^. That genes facilitating maintenance of a normal microtubule network are downregulated in SSP treatments is a characteristic of an orderly form of PCD. The absence of this downregulation during Zt6 expression is consistent with a form of cell death relying less on cytoskeleton arrangement. In contrast, GO term analysis revealed enrichment of terms associated with ribosome, structural constituent of the ribosome, translation and translation initiation factor activity in genes upregulated by Zt6 at 48hpi (Table 2). This suggests Zt6 treatment induced a significant stress on ribosome function and on protein translation in general. These terms are not enriched in either up- or downregulated groups for the SSP treatments at either timepoint (Tables 4, 5). An upregulation of genes related to ribosome structure and function could be indicative of cells experiencing ribosomal stress and therefore perturbations in protein synthesis. This would be anticipated in cells expressing Zt6 which has previously been shown to cleave plant rRNA in a semi-specific manner^20^. These expression signatures are a likely response to compensate for reduced ribosome functionality in cells expressing Zt6.

The GO terms protein kinase activity and protein serine/threonine kinase activity were strongly induced by Zt6 treatment at the 24hpi timepoint (Table 2). In contrast, these groups were unchanged at 24hpi and subsequently downregulated at 48hpi in the SSP treatment group (Table 5). The activity of several serine/threonine kinase proteins is important for control of apoptosis and autophagy in animal systems^44^. However, the role of this protein class in direct activation of plant cell death is less clear. Many transmembrane receptor kinases and intracellular kinases play important roles in ligand perception and signal transduction during pathogen interaction. We were specifically interested in expression patterns of genes belonging to the RLP, RLK and WAK receptor families. These classes of receptors are well known to be involved in recognition of PAMPs or apoplastic effectors. Indeed, the only two cloned R genes against *Z. tritici* are *Stb6* and *Stb16q*, encoding a WAK and a CRK respectively^24,45^. Expression of immune receptors is often upregulated in response to pathogens^23–25^. Here, we found a number of genes annotated as receptor-like kinases, WAKs, LRR-RLKs, CRKs and LecRLKs were transcriptionally responsive to SSP expression (Fig. 4). Expression patterns were similar between SSP effector treatments in comparison to Zt6. Most differentially expressed receptors in these classes were downregulated upon SSP expression, but upregulated in response to Zt6. A small number of genes annotated as NLRs were induced by Zt6 expression only. A larger number of genes annotated as RLPKs were differentially expressed upon SSP treatment. Again, there is clear differentiation between expression patterns induced by SSPs in comparison to Zt6. Nearly all RLPKs differentially expressed in the Zt6 group were upregulated, whereas nearly all RLPKs that changed in response to SSPs were downregulated. Our data therefore provides a small number of candidate cell surface immune receptors for recognition of *Z. tritici* SSPs for validation in a follow-on study.

The GO terms proteasome core complex and proteolysis involved in cellular protein catabolic process were enriched amongst upregulated genes for all three of the SSP group treatments at 48hpi. In contrast, proteolysis was enriched amongst downregulated genes at 48hpi for the Zt6 treatment. The contribution of proteasome function to PCD has previously been investigated. Silencing of components of the 26S proteasome leads to build-up of polyubiquitinated proteins and induction of PCD^46^, suggesting proteasome function negatively regulates PCD. In contrast, Hatsugai and colleagues identified a mechanism involving PBA1 linking proteasome function with promotion of PCD^47^. These contrasting results suggest that role of the proteasome in cell death may be highly complex.

Given the differences in expression profile of many receptor gene groups between Zt6 and SSP treatments, we focussed on TF family expression patterns. Overall, Zt6 expression led to induction of many NAC, MYB and WRKY TFs (Fig. 6). In contrast, the majority of these genes were either unresponsive or repressed by SSP expression. This pattern was also true for TCP TFs, although the number of genes that were differentially expressed were much lower. NAC TFs play important roles in senescence and in both biotic and abiotic stress responses. Expression of the NAC transcription factor gene *Niben101Scf01498g04003* was striking as it was strongly upregulated in the Zt6 48 h treatment exclusively (Fig. 6A). This gene shows a high level of homology to *A. thaliana ANAC032* (*E*-value = 8e−08). *ANAC032* regulates senescence through modulation of *AtNYE1*, the so-called *STAY-GREEN* gene involved in ability to catabolize chlorophyl^38^. In *N. benthamiana* there are six *STAY-GREEN* genes, however, only two were responsive to treatment and this pattern was similar across Zt6 and SSP treatments. This suggests that while transcriptional promotion of senescence at the level of TF genes appears starkly different between Zt6 and SSP treatments, there may yet be some downstream regulatory convergence.

Other pathways by which upregulation of *Niben101Scf01498g04003* may promote senescence are alluded to by its other closest homologs in *A. thaliana*. The second of these was *ANAC047* (AKA. *SPEEDY HYPONASTIC GROWTH*) (*E*-value = 1e−07). *ANAC047* is upregulated during and may promote leaf senescence via regulation of *ACO5* (1-aminocyclopropane-1-carobxylic acid oxidase 5), an enzyme involved in ethylene biosynthesis^48,49^. In our results, a number of genes annotated as *ACO5* and *ACO5* homologs exhibit both up and downregulation in the Zt6 treatments. Alternatively, the 10^th^ closest homolog of *Niben101Scf01498g04003* is *ANAC082* (*E*-value = 2e−05) which plays a role in the sensing of nucleolar stress^50^. This is due to the presence of an upstream open reading frame (uORF) in *ANAC082* mRNA. Previously studied uORFs act as negative regulators of the main ORF due to ribosome stalling on the mRNA. Upregulation of *ANAC082* expression could therefore be indicative of ribosome instability induced by Zt6 expression. Given the known interaction between Zt6 and rRNA, this could provide a mechanism for Zt6-specific patterns of transcriptional reprogramming and a role for this TF as a master regulator of downstream gene expression.

A number of MYB TF genes were also highly upregulated by Zt6 but not by other treatments (Fig. 6B). Of these, *Niben101Scf04560g07009* and *Niben101Scf01694g12010* were most strongly induced. These genes are orthologous to *AtMYB36/MYB68* and *AtMYB119* respectively. These TFs are known to have roles in root cell differentiation^51^, root development^52^ and cellular differentiation during female gametogenesis^53^. However, these TFs are not documented to be involved in induction of cell death or pathogen responses. Similarly, the *N. benthamiana* ortholog of *AtTCP5* was induced by Zt6 treatment alone (Fig. 6C). This TF has roles in floral development and ethylene biosynthesis^54^ but not known to be involved in cell death pathways.

WRKY transcription factors have been identified as important regulators of biotic stress responses^55^. It is possible that increased WRKY activity accounts for the modified expression of defence genes such as RLPs and RLKs. Therefore, the complex interplay between these TF families might explain the transcriptional profile induced by the effector treatments. A number of WRKYs were transcriptionally induced by Zt6 but not by SSP expression (Fig. 6D). Of these, *Niben101Scf01281g05001* and *Niben101Scf12560g00018* were the most strongly upregulated. These TFs are orthologues to *AtWRKY14/35* and *WRKY22* respectively. Constitutively activated WRKY14 is known to promote cell death in *N. benthamiana*^56^. *WRKY22* has previously been implicated in plant defence responses and loss of *WRKY22* expression compromises effector-induced cell death in *N. benthamiana*^57^. This suggests that WRKY-dependent transcriptional reprogramming may contribute to cell death induced by Zt6.

Taken together our results show that *Z. tritici* secreted ribonuclease Zt6 and three SSPs, trigger cell death in non-host *N. benthamiana* at least partially via gene expression changes in clearly distinct cohorts of genes. Within these cohorts, only a small number of genes that could function as immune receptors were upregulated in response to SSP expression, and therefore provide a manageable set of candidates for further study as potential *Z. tritici* non-host R-genes. Among TF genes in these cohorts, those transcriptionally responsive to Zt6 expression suggest a potential pathway to ribosomal stress induced PCD. This work provides a detailed picture of transcriptional changes that occur in *N. benthamiana* prior to cell death induced by apoplastic recognition of non-host pathogen SSPs, and ribonuclease activity.

## Methods

### Plants and bacterial strains

All *N. benthamiana* plants were from a seed stock used in our previous investigation under the same growth conditions^19,20^. The *Agrobacterium tumefaciens* GV3101 (pMP90) strains expressing Zt6, Zt9, Zt11 and Zt12 from pEAQ-HT-DEST3 were described previously^19,20^.

### Generation of RNA samples

Leaves of 5-week old *N. benthamiana* seedlings were syringe infiltrated with Agrobacterium suspensions at OD_600_ = 1.2 in Agroinfiltration buffer (10 mM MgCl_2_, 10 mM MES, 150 µM acetosyringone, pH 5.6). Six plants were infiltrated per treatment with 30 plants used in total. Leaf sampling was performed at 24 h and 48 h post infiltration. Three infiltrated leaf patches, one each from three individual plants were cut from leaves and pooled to produce each sample. Samples were snap frozen in liquid nitrogen and stored at − 80 °C until processing.

### RNA extraction

Frozen leaf samples were ground in liquid nitrogen using a mortar and pestle. RNA was extracted using a Trizol/Chloroform procedure as described previously^58^. DNase digest was performed using RQ1 DNase (Promega) and RNA recovered by ethanol precipitation. RNA quality and purity was measured using Qubit and Nanodrop. RNA-seq was performed by BGI on the Illumina HiSeq2500 platform.

### Quality control and alignment

Quality of raw reads was manually assessed using FastQC software v0.119^59^. Filtering of raw reads was then performed using PRINSEQ-lite software v0.20.4 to a minimum Phred-quality score of 26^60^. Version 1.0.1 of the *N. benthamiana* reference genome was downloaded from the Sol Genomics Network ftp site (ftp://ftp.solgenomics.net/genomes/Nicotiana_benthamiana). Index of the reference genome was built using the build function in HISAT2 v2.1.0, filtered paired-end reads were aligned to the reference genome using the same software^61^. Following alignment, resulting .BAM files for each treatment were checked for uniformity of gene body coverage and sufficient reads per kilo base per million mapped reads (RPKM) saturation using RSeQC v2.6.4^62^.

### Read counting and differential expression analysis

Reads mapped to each gene were counted using HTSeq v0.11.0. Differential expression analysis was conducted between each treatment and the control treatment (GFP) at the appropriate time point using the DESeq2 R package v1.32.0, with a Benjamini-Hochberg – false discovery rate (BH-FDR) corrected *P*-value of 0.05^22^.

### Gene ontology enrichment analysis

Significantly differentially expressed genes (DEGs) were divided into those that were up-regulated and those that were down-regulated at each time point in each treatment. Gene ontology enrichment analysis was conducted on each of these two groups of DEGs using the GOseq R package with BH-FDR *P*-value adjustment and gene length bias correction, GO terms with adjusted *P*-values less than 0.05 were considered to be significantly enriched in that group^22^.

### Ethics approval

All handling of plants, microorganisms and associated samples in this study was performed under biosafety regulations in place at Rothamsted Research. All work was carried out under the Department for Environment, Food and Rural Affairs (Defra) plant health licenses Nos. 101941/197343/8 and 101948/198285/4.

## Supplementary Information

Supplementary Information 1.

Supplementary Information 2.

## Data Availability

All RNA-seq raw sequencing data used in this study were deposited into the NCBI SRA under BioProject accession number PRJNA858969.
