# Supplementary material for: Induction of distinct plant cell death programs by secreted proteins from the wheat pathogen Zymoseptoria tritici
Source: Sci Rep. 2022 Oct 25;12:17880. doi: 10.1038/s41598-022-22660-9 (PMC9596407; doi:10.1038/s41598-022-22660-9)
Supplement: Supplementary file 2 — Supplementary Information 2. [file 41598_2022_22660_MOESM2_ESM.docx]

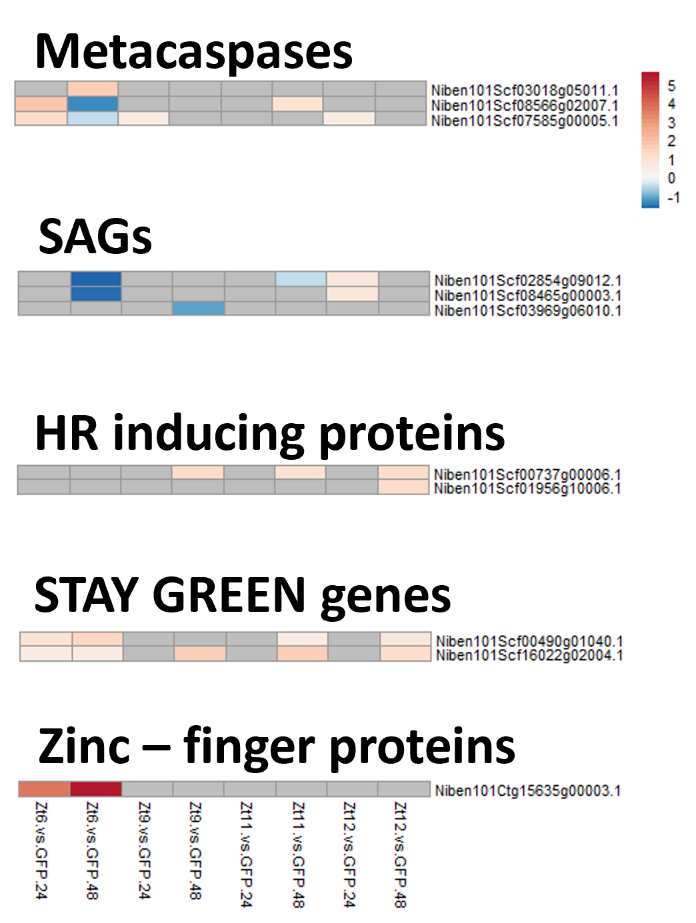


Supplementary figure 1: Expression profiles of non-TF senescence regulating genes with marked difference in expression change between Zt6 and Zt9, Zt11, and Zt12.
